# Applying a non-GMO breeding approach with an identified natural variation to reduce food allergen Len c3 in *Lens culinaris* seeds

**DOI:** 10.3389/fpls.2024.1355902

**Published:** 2024-04-12

**Authors:** Jingpu Song, Ioannis Mavraganis, Wenyun Shen, Hui Yang, Jitao Zou

**Affiliations:** Aquatic and Crop Resource Development Research Centre, National Research Council of Canada, Saskatoon, SK, Canada

**Keywords:** LTP, Len c3, lentil allergen, legume, natural variation

## Abstract

Lentils (*Lens culinaris*) are produced in diverse agroecological regions and are consumed as one of the most important food legumes worldwide. Lentils possess a nutritional profile from a human health perspective that is not only nutrient dense but also offers a better balance between protein and carbohydrates. However, lentil causes food allergy, which has been a significant concern due to increased consumption in parts of the world. Len c3, a non-specific lipid transfer protein (LTP), was identified as one of the allergens in lentil seeds. In this study, we identified an LTP gene *Lcu.2RBY.4g013600* that encodes the lentil allergen Len c3. We then focused on gene screening from a collection of natural accessions to search for natural mutations of the Len c3 allergen-encoding gene. A natural lentil line M11 was identified with mutations at *LcLTP3b* and low accumulation of vicilin through genomic-assisted approaches. Furthermore, we generated a pool of lentil germplasms with *LcLTP3b* mutation background through crossing the identified lentil plant M11 with two lentil cultivars, CDC Redmoon and CDC Gold. These generated lentil hybrids can be used as a breeding resource targeting at reducing allergen risk in lentil consumption.

## Introduction

Lentils (*Lens culinaris*) have nutritional, economical, and environmental advantages as an excellent source of protein-based human diet (Kumar et al., 2016). Lentils have twice the level of proteins than cereals and are rich in minerals, polyphenols, and vitamins (Yu et al., 2023). Lentil proteins have been a source of novel food formulations in milk substitute, curd-like products, meat products, extruded products, and baked goods (Boye et al., 2010). Notwithstanding its superb nutritional characteristics, consumption of lentil proteins causes food allergy among some individuals, particularly in pediatric population.

Lentils are the major causes of IgE-mediated allergic reactions in children of the Mediterranean population (Pascual et al., 1999). In Spain, allergy to lentils ranks the fifth most common cause of IgE-mediated food allergy in children (Martínez San Ireneo et al., 2008; Valenta et al., 2015). In Turkey, where most of the populations consume legumes as staple food, lentils are the sixth most common food allergen in the pediatric population as well (Akarsu et al., 2021). Similarly, in India, where legumes are essential protein sources of vegetarian diet, lentils and chickpeas are recognized as key contributing factors to legume allergy (Patil et al., 2001). Three major allergen groups from lentils have been identified. Len c1 is derived from vicilin, the most abundant component of seed storage protein (López-Torrejón et al., 2003). Len c2 is a 66-kDa biotinylated protein (Sánchez-Monge et al., 2000), and Len c3 is a non-specific lipid transfer protein (LTP) of approximately 9 kDa (Akkerdaas et al., 2012). Len c3 was extracted from germinated lentils seeds and verified as immunologically potential allergens using immunoblot analysis (Akkerdaas et al., 2012). LTPs can easily bind to multiple types of lipid molecules such as fatty acids and phospholipids (Shenkarev et al., 2017). Although Len c3 is less abundant compared with Len c1 and Len c2, LTPs are highly cross-reactive and are considered as one of the main plant allergens (Shenkarev et al., 2017; Halima et al., 2022).

While crop breeding and agronomic advances have greatly increased crop yield, food security on a global scale urgently demands improvements in nutritional quality. Natural variations harbor numerous mutations and abundant historical recombination and are cost effective for population development phenotyping and repeated phenotyping (Liang et al., 2021). A major challenge is to identify and utilize the advantageous traits in a breeding program (Gur and Zamir, 2004). Molecular tools for lentil breeding such as genome sequencing and transcriptome profiling are being developed rapidly (Fedoruk et al., 2013; Haile et al., 2020; Song et al., 2022; Yu et al., 2023), which have rendered this relatively facile for identifying the target genes. In this study, we identified the LTP gene that encodes Len c3 in lentil and performed genetic screening via gene sequencing for LTP mutation from a collection of natural lentil accessions. We further conducted transcriptome analysis of developing seeds to investigate gene expression of vicilin in the identified natural variation. Lastly, the identified natural *LTP* mutation was introduced into two cultivated lines, CDC Redmoon and CDC Gold, to generate an allergen-less germplasm pool.

## Materials and methods

### Plant materials and growth conditions

Plants were grown in a growth chamber under 16 h light, 23°C and 8 h dark, 18°C, with far red light for flowering. Lentil seeds, collected individually from 400 natural accessions (**Supplementary File 1**) originated from the Mediterranean regions, were received from Plant Gene Resources of Canada (PGRC). Two lentil cultivated lines, CDC Redmoon and CDC Gold, were also included in this study.

### Phylogenetic analysis

Lentil LTP peptide sequences were obtained from lentil genome assembly v2.0 (Ramsay et al., 2019). The information on Len c3 was found on Allergome (www.allergome.org), and the peptide sequence of Len c3, shown as LTP2, was downloaded from NCBI (accession no. A0AT29.1). Nucleotide and peptide sequences were aligned by using MEGA 11 software (Tamura et al., 2021), and phylogenetic tree was generated by using online tool iTOL (Letunic and Bork, 2021).

### *LcLTP3b* gene cloning and sequence alignment

Genomic DNA of each lentil line was isolated from lentil leaves. *LcLTP3b* gene amplification was conducted by using Phusion DNA polymerase (New England Biolabs, MA, USA). The amplified PCR products were purified by using a PCR Purification Kit (Qiagen, Canada) before Sanger sequencing. The sequencing results were analyzed by using MegAlign Pro software.

### Identification of *lcltp3b* allele

To whether the hybrid plants harbor the *lcltp3b* allele, the first exon of *LcLTP3b* gene was cloned and subjected to Mse1 treatment for 30 min before agarose gel electrophoresis.

### Storage protein analysis

Seed storage protein isolation and separation was performed as described previously (Song et al., 2021). Briefly, three seeds of each lentil line were ground, and 0.1 g was used for protein isolation. Protein samples were separated on a 15% SDS-PAGE gels. After separation by electrophoresis using a Biochrom Novaspec Plus Visible Spectrophotometer (Bio-RAD), the protein gels were stained with Coomassie Brilliant Blue R250 for 30 min, followed by de-staining for 1 h with de-staining solution before imaging with ChemiDoc Imaging System (Bio-RAD).

### RNA extraction and data analysis

Total RNA was extracted from dissected embryos and seed coats containing endosperms using a RNeasy plant mini kit (Qiagen, Germany) according to the manufacturer’s instruction. For transcriptome sequencing, cDNA libraries were constructed from the isolated RNA samples by using a TruSeq RNA Sample Preparation kit v2 (Illumina). The cDNA libraries were used for RNAseq. RNAseq was conducted on an Illumina NOVAseq 6000 pair-end sequencing. RNAseq data analysis was conducted as previously described (Song et al., 2022).

### Seed imaging and analysis

Seed images were taken using a Canon EOS70D with a MACRO 100 mm lens. Seed diameters were determined by Image J.

### Data availability

RNAseq data that support the findings of this study have been deposited in the Gene Expression Omnibus under accession code GSE255951.

## Results

### *LcLTP3b* encodes the allergen Len c3 in lentil

**Figure 1:**
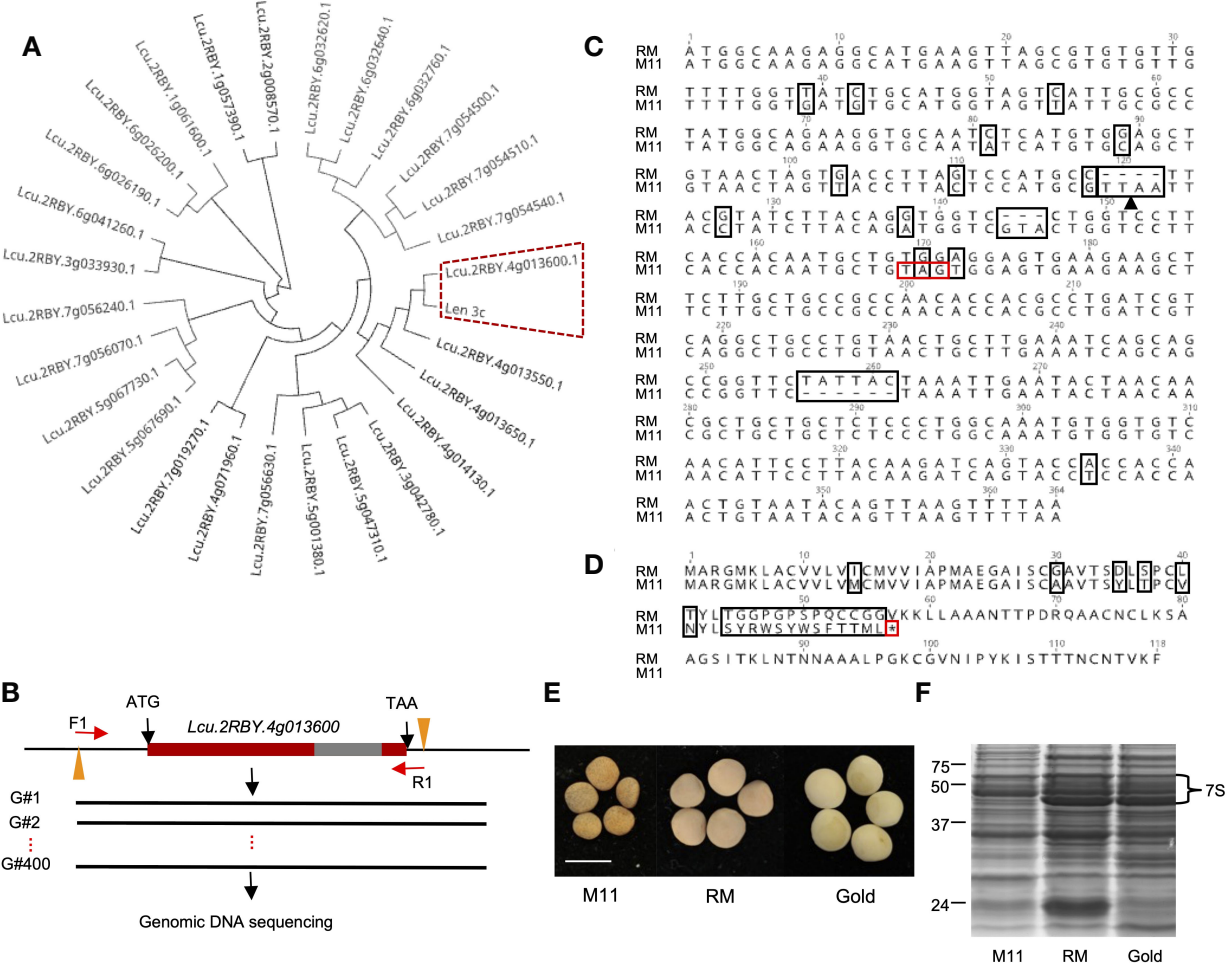
Identification of natural lentil line harboring mutated allele of *LTP* gene that encodes Len c3 allergen. **(A)** Phylogenetic analysis of lentil LTP peptides and Len c3. Len c3 and its closest lentil LTP are highlighted with red dash lines. **(B)** Schematic workflow showing the genetic screening on 400 wild lentil lines. Red bars indicate exon, and gray bar represents intron. F1, forward primer; R1, reverse primer. **(C)** Nucleotide sequence alignment of *LcLTP3b* sequenced from CDC Redmoon (RM) and the natural mutation line 11 (M11). All alignment disagreements are highlighted in black boxes; black arrow indicates Mse1 restriction site; the induced stop codon is highlighted with a red box. **(D)** Peptide sequence alignment of LcLTP3b translated from RM and M11. All alignment disagreements are highlighted in black boxes. The induced translation termination site is highlighted with a red box. **(E)** Image of mature seeds collected from M11, RM, and CDC Gold (Gold). Scale bars: 5 mm. **(F)** SDS-PAGE gel image showing the 7S vicilin in seeds from M11, RM, and Gold.

To identify the LTP that encodes Len c3, we first conducted a phylogenetic analysis of the known 26 lentil LTP peptide sequences and Len c3. The phylogenetic tree showed that Lcu.2RBY.4g013600 and Len c3 were grouped together (**Figure 1A**), and further sequence alignment result indicated that they were 100% identical (**Supplementary Figure S1**). We concluded that lentil LTP gene *Lcu.2RBY.4g013600* (*LcLTP3b*) encodes the allergen protein Len c3.

### Identification of natural lentil germplasm with *LcLTP3b* mutation

To perform a genomic screening of a collection of natural lentil germplasms, we first retrieved the genomic DNA sequence of *LcLTP3b* from the lentil genome. Next, we designed a pair of primers: the forward primer (F1: CTAACACCCGTTAAGACATTGC) was at ~100 bp upstream of the translation start site (ATG) and the reverse primer (R1: ATAGCCTTGGAACCGCAACA) was at ~20 bp downstream of the stop codon (TAA) (**Figure 1B**). Furthermore, we cloned the *LcLTP3b* gene from the 400 lentil lines, respectively, followed by Sanger sequencing. Sequencing results were aligned to the *LcLTP3b* sequence. Among the collected lentil accessions, we identified one line “CN45073” (**Supplementary File 1**) with several mutations at *LcLTP3b*, including two insertions, one deletion, and one stop-gain point mutation (**Figures 1C, D**). This lentil line was referred as M11 in this study, and the mutated gene was marked as *lcltp3b*.

### Low vicilin accumulation in the M11 seeds

**Figure 2:**
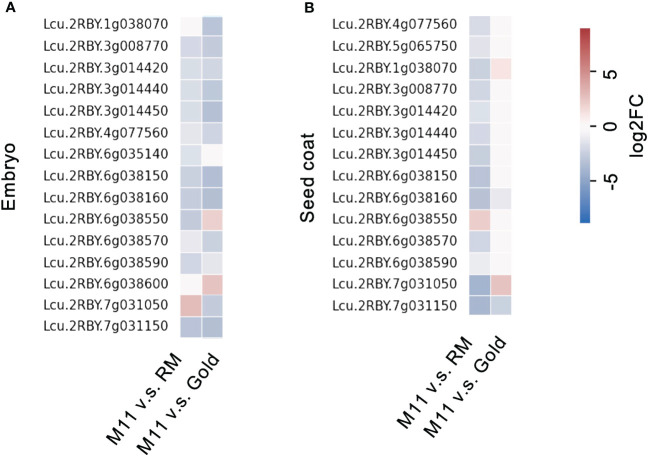
Fold change of transcription levels of seed storage protein genes encoding 7S vicilin in the dissected mature green seeds (**A**, embryo; **B**, seed coat with endosperm) of M11 compared to RM and Gold.

Lentil seed storage protein quantity and yield are important agronomic traits in lentil breeding (Subedi et al., 2021). Next, we investigated the seed storage protein levels of the identified M11 seeds. We isolated total proteins from M11 mature dry seeds and two CDC (Crop Development Centre) cultivars, CDC Redmoon (Redmoon, hereafter), and CDC Gold (Gold, hereafter) (**Figure 1E**) and conducted seed storage protein analysis via SDS-PAGE electrophoresis. The results indicated that M11 seeds had much lower 7S protein level compared to Redmoon and Gold (**Figure 1F**). 7S proteins are enriched with vicilin where another lentil allergen *Len c1* is derived (López-Torrejón et al., 2003). Thus, we performed transcriptome analysis of the mature green seeds to investigate the expression levels of seed storage protein genes encoding 7S vicilin. In seed embryos, the majority of 7S vicilin encoding genes in M11 exhibited lower expression levels compared to Redmoon and Gold, while in the seed coat attaching endosperm tissues, M11 had lower expression levels compared to Redmoon, but higher than that of Gold (**Figure 2**, **Supplementary File 2**). Seed storage proteins are mainly accumulated in seed embryos. Overall, these results indicated that M11 had lower accumulation levels of 7S vicilin compared to the other two cultivars.

### Creation of a pool of lentil hybrids with allergen-less traits

**Figure 3:**
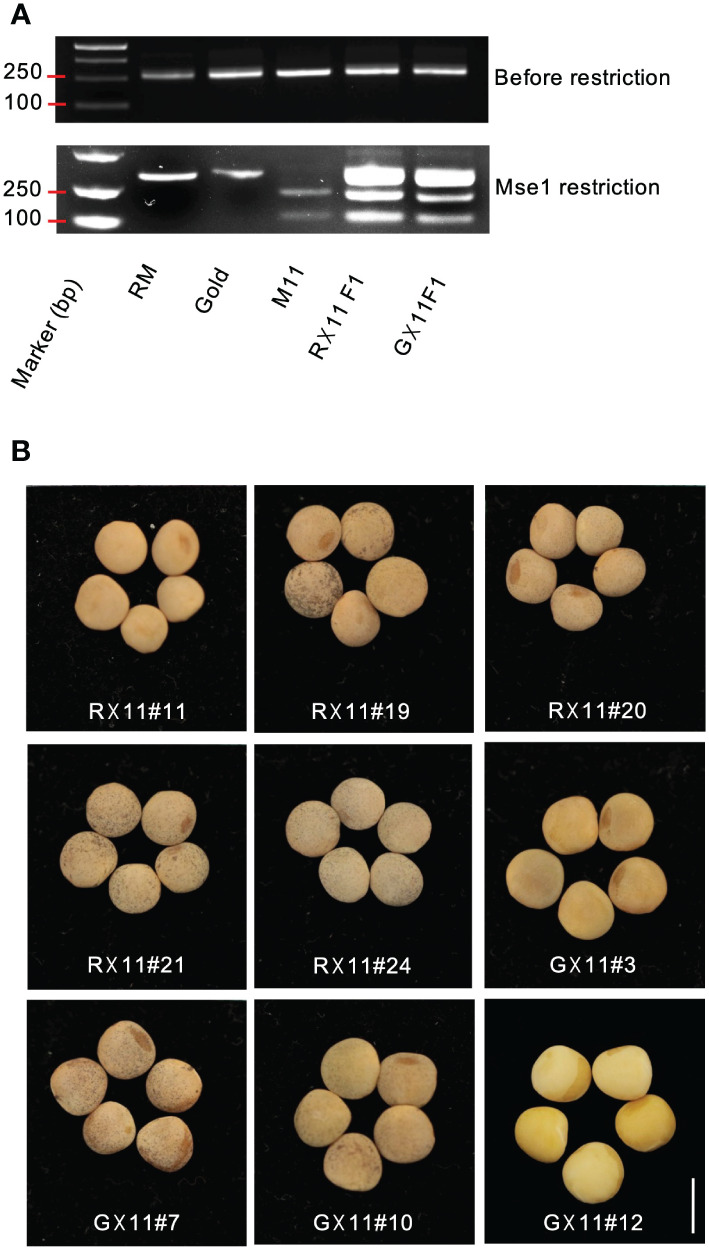
Identification of the hybrid lentil lines generated from the crosses of Redmoon×M11 (R×11) and Gold×M11 (G×11). **(A)** PCR and Mse1 restriction-based genotyping for lentil hybrid identification. **(B)** Lentil seeds collected from the F2 plants of R×11 and G×11 harboring homozygous *lcltp3b*. Scale bars: 5 mm.

To incorporate the mutation allele *lcltp3b* into commercial cultivars, we pollinated M11 with pollen grains collected from Redmoon and Gold, respectively. Hybrids of Redmoon × M11 (R×11) and Gold × M11 (G×11) were produced and identified by PCR-based genotyping. We first amplified the full-length genomic DNA of *LcLTP3b* gene with the first set of primers (F1, CTAACACCCGTTAAGACATTGC; R1, ATAGCCTTGGAACCGCAACA) from the plant genomic DNA. Of note, the first insertion site located at the first exon of *lcltp3b* created a Mse1 restriction site. Thus, we took advantage of this mutation site and designed a second set of primers (F2, ATGGCAAGAGGCATGAA; R2, TTAGAAAAAGACATACGTATTAC) to clone only the first exon of *LcLTP3b (LcLTP3b-exon)* by using the genomic DNA of *LcLTP3b* gene as template. After Mse1 treatment on the amplified PCR products, *lcltp3b-exon* originated from M11 was cut into two fragments, while *LcLTP3b-exon* obtained from either Redmoon or Gold remained intact (**Figure 3A**). We further propagated the second generation to screen for the *lcltp3b* homozygous lines. The *lcltp3b* homozygous lines (five R×11 lines and four G×11) were identified by using the same PCR-based enzyme restriction assays, and their mature seeds were collected, separately (**Figure 3B**). In addition, agronomic traits of seeds from these lines are documented in **Table 1**. Taken together, we have successfully incorporated the natural mutation *lcltp3b* into other lentil cultivars and generated a pool of lentil germplasms.

**Table 1:** Agronomic traits of Redmoon, Gold, 11 and hybrid seeds.

Genotype	Diameter (cm)	Weight (mg)	Seed coat	
			Color	Dark spots
CDC Redmoon	0.49 ± 0.02	49.0 ± 1.3	red	no
CDC Gold	0.53 ± 0.04	53.6 ± 3.8	golden	no
Mutant(11)	0.41 ± 0.04	37.8 ± 1.2	red	yes
Rx11#11	0.46 ± 0.04	46.0 ± 2.6	red	no
Rx11#19	0.53 ± 0.02	52.7 ± 4.0	red	yes
Rx11#20	0.48 ± 0.02	46.7 ± 1.3	red	yes
Rx11#21	0.50 ± 0.02	51.9 ± 2.8	red	yes
Rx11#24	0.50 ± 0.02	46.0 ± 0.8	red	yes
Gx11#3	0.51 ± 0.02	48.5 ± 3.7	golden	yes
Gx11#7	0.54 ± 0.01	59.0 ± 4.3	red	no
Gx11#10	0.55 ± 0.01	53.1 ± 1.2	red	yes
Gx11#12	0.56 ± 0.01	64.1 ± 2.6	golden	no

## Discussion

Allergens in lentil have been a significant concern due to increased consumption in parts of the world (Sackesen et al., 2020). Numerous efforts have been made to identify and qualify lentil allergens, but lentil allergy management through modifications in food systems requires further development (Halima et al., 2022). One goal in crop breeding for better seed nutrition quality is to reduce levels of anti-nutritional factors, such as allergens (Song et al., 2022). In this study, we took a non-GMO approach, focusing on exploring natural variations to search for lentil accessions that harbor natural mutated allergen-encoding genes.

Up to date, three major allergens are known in lentil seeds. Although Len c3 is a rather minor component of lentils’ seed protein portfolio, its interacting IgE is detected in 9 out of 10 patients’ sera, indicating its prevalence in invoking allergic responses (Akkerdaas et al., 2012). LTPs are a group of plant proteins initially defined by their ability to bind polar lipids in a non-specific manner *in vitro* (Salminen et al., 2016). Voluminous literature indicates that sensitization to LTPs can lead to cross-reaction to homologous food allergens (Rial and Sastre, 2018). From a plant productivity point of view, LTPs represent a minuscule portion of total seed proteins in lentils. Hence, eliminating LTPs will not affect seed protein yield or nutritional profiles. In this study, Len c3-encoding gene *LcLTP3b* was first identified and used as a target for genetic screening to identify natural mutations in a large collection of natural accessions (**Figures 1A, B**). The screening process identified one natural variation, named M11, harboring mutation at *LcLTP3b*, naming *lcltp3b* (**Figure 1C**). A previous study has shown that mutations at Thr41, Arg45, and/or Tyr80 significantly affect the ligand-binding capacity and the allergenic potential of Len c3 (Melnikova et al., 2021). The *Lcu.2RBY.4g013600* gene encodes a functional 118-aa (amino acid) protein, while the mutated gene only encodes a 56-aa abnormal protein with substitutions at sites 40/45 (**Figure 1D**), suggesting that the mutation of *Lcu.2RBY.4g013600* reduces allergens in M11. Moreover, the identified lentil variation M11 has a much lower vicinlin (7S seed storage protein) level, which might be due to a lower transcription activity (**Figures 1F**, **2**) compared to two commercial cultivars, CDC Redmoon and CDC Gold. Taken together, the genetic evidence suggests that M11 appear to produce seeds containing less allergens.

For breeding purpose, we introduced the mutated allele *lcltp3b* from M11 into Redmoon and Gold, which are in different genetic backgrounds through genetic crosses. We have obtained *lcltp3b* homozygous lines from the two crosses, five lines from the Redmoon × M11 crosses and four lines form the Gold × M11 crosses (**Figure 3B**). The F2 generation lines produced seeds varying in seed size, weight, and seed coat color (**Table 1**). However, these agronomic traits need continuous breeding process to be fixed. It would be useful to develop a haploid inducer line to shorten the stabilization process of these *lcltp3b* homozygous lines (Gilles et al., 2017; Chen et al., 2023). The *lcltp3b* homozygous lines are the results of different genetic combinations of M11 and other cultivars; thus, they can be directly used as breeding materials in searching for favorable agronomic traits in in the allergen-less background. The findings of this study reveal the potential of lentil natural variation M11 as a breeding material for reducing allergy risk.

## Data availability statement

The original contributions presented in the study are included in the article/**Supplementary Materials**, further inquiries can be directed to the corresponding authors.

## Author contributions

JS: Investigation, Methodology, Writing – original draft. IM: Formal Analysis, Methodology, Software, Writing – review & editing. WS: Methodology, Writing – review & editing. HY: Methodology, Writing – review & editing. JZ: Conceptualization, Project administration, Supervision, Writing – original draft.
