# Supplementary material for: Applying a non-GMO breeding approach with an identified natural variation to reduce food allergen Len c3 in Lens culinaris seeds
Source: Front Plant Sci. 2024 Apr 12;15:1355902. doi: 10.3389/fpls.2024.1355902 (PMC11090098; doi:10.3389/fpls.2024.1355902)
Supplement: Supplementary file 1 [file Presentation_1.pdf]

Supplemental Fig.1

```
a Len c3 length:118
b Lcu.2RBY.4g013600 length:118
  Alignment length: 118
  Identity:     118/118 (100.00%)
  Similarity:   118/118 (100.00%)
  Gaps:           0/118 (0.00%)

a1    M A R G M K L A C V V L V I C M V V I A P M A E G A I S C G A V T S D L S P C L T Y L T G G P G P S P Q C C G G V K K L
      | | | | | | | | | | | | | | | | | | | | | | | | | | | | | | | | | | | | | | | | | | | | | | | | | | | | | | | | | | | |
b1    M A R G M K L A C V V L V I C M V V I A P M A E G A I S C G A V T S D L S P C L T Y L T G G P G P S P Q C C G G V K K L

a61   L A A A N T T P D R Q A A C N C L K S A A G S I T K L N T N N A A A L P G K C G V N I P Y K I S T T T N C N T V K F
      | | | | | | | | | | | | | | | | | | | | | | | | | | | | | | | | | | | | | | | | | | | | | | | | | | | | | | | | | |
b61   L A A A N T T P D R Q A A C N C L K S A A G S I T K L N T N N A A A L P G K C G V N I P Y K I S T T T N C N T V K F
```

Supplemental Fig. 1 Peptide sequence alignment of Len c3 (a) and *Lcu.2RBY.4g013600*-encoding LTP (b).
